# Effectiveness of innovative chest compression on the emergency department stretcher by an alternative method: a randomized controlled crossover trial

**DOI:** 10.1038/s41598-024-62845-y

**Published:** 2024-05-29

**Authors:** Nawaphon Charungwatthana, Pavita Laohakul, Theerapon Tangsuwanaruk, Borwon Wittayachamnankul

**Affiliations:** Department of Emergency Medicine, Faculty of Medicine, Chiang Mai University, 110 Inthawaroros Road, Sribhumi, Amphoe Muang Chiang Mai, Chiang Mai, 50200 Thailand

**Keywords:** Cardiopulmonary resuscitation, Chest compression, Rescuer's position, Cardiology, Public health

## Abstract

Comparison of the three methods standing-on-a-stool (SS), one knee kneeling on a stretcher (KS), and kneeling at the same height as the patient’s bed on the kneeling stool (KK) to evaluate the yielded of highest CPR quality and rescuer comfortability. A prospective randomized cross-over study which compares the three different rescuer positions for their effectiveness of chest compression. Conducted at a tertiary care between 19 and 22 Aug 2022. Emergency personnel aged 18 years or older, who completed the AHA-approved BLS course**.** The chest compression data was obtained by the ALIVE AED manikin® and AED reporting system. The information on the CPR quality and post-test questionnaires assessing the participants’ preference, satisfaction and modified Borg’s scale score was collected. The three methods shown no statistically significant difference in adequate chest compression depth (percentage). KK was not superior than SS at chest compression rate (P = 0.05). The adequate full chest recoil achieved with KK and KS were significantly higher than that of SS. However, there were no statistical difference between the three methods in the modified Borg’s scale score. Based on the satisfying score, the rescuers preferred KK to either SS (p 0.007) or KS (p 0.012). The three methods shown no statistically significant difference in adequate chest compression depth (percentage). Still, both KK and KS provided more adequate chest recoil, so we would recommend using these two methods in the ED.

**Clinical trial registry:** This study was retrospective registration in thaiclinicaltrials.org (TCTR20230119002, 19/1/2023).

## Introduction

High-quality cardiopulmonary resuscitation (CPR) is essential for improving the survival of Out of Hospital Cardiac Arrest (OHCA); in emphasizing high-quality CPR, the compression depth should be at least 5 cm but not exceed 6 cm for adult cardiac arrest patients. The chest should be released and allowed to recoil completely before initiating another compression, and the compression rate should be at least 100–120 compressions per minute. It is crucial to ensure that no interruptions occur while performing chest compression^1–3^.

The layperson and hospital staff may learn basic life support (BLS) in various programs, enabling them to become BLS providers and feel confident in emergencies situation^4–6^. However, the American Heart Association (AHA) guidelines and the European Resuscitation Council (ERC) guidelines do not provide any details about the rescuer’s position and posture during in-hospital cardiac arrest, especially in emergency department (ED) on stretcher that is different from the practice in BLS lesson except for the using a rigid backboard on the patients' backs. According to the literature review, kneeling positions were effective on a hard floor or on a wide patient bed (with space for kneeling beside the patient). Stand-on-a-stool is an effective method that shows non-inferiority to kneeling beside the patient; the principle is that the rescuer's knees are equally close to the patient's bed^7–9^. Standing on a stool has some disadvantages; this method can cause low back pain and imbalance^10,11^, and kneeling is impossible in the ED.

As a result, we developed an innovation that can simulate a rescuer in a kneeling position, the kneeling stool. Suppose stability is one of the reasons for effective chest compression. In that case, we included another method in which the rescuer placed one dominant knee on the bed and performed the chest compression because this posture is simple and brings the rescuer’s knee level with the patient’s bed. Hence, we aimed to demonstrate which of the three methods: stand-on-a-stool (SS), one knee kneeling on a stretcher (KS), and kneeling at the height as the patient’s bed on the kneeling stool (KK), was the best way to improve CPR quality and rescuer comfortability.

## Methods

### Trial design and setting

This study is a crossover, open-label, single-centered, randomized controlled trial conducted at Chiang Mai University Hospital, a tertiary care, university-based hospital between 19 AUG 2022 and 22 AUG 2022 to compare the effectiveness of chest compression in three different rescuer positions: kneeling on the kneeling stool (intervention), stand-on-a-stool, and one knee kneeling on a stretcher and standing on a step stool.

The study was conducted in accordance with the principles outlined in the Declaration of Helsinki, and the study protocol received exempt approval from the Research Ethics Committee of the Faculty of Medicine, Chiang Mai University (study code: EME-2564-08366). This study followed the Consolidated Standards of Reporting Trials (CONSORT) 2010 statement with extension to randomized crossover trials. Each participant provided written, informed consent before enrollment.

### Participant

We recruited emergency personnel aged 18 years or older, including nurses, doctors, paramedics, and nurse aids who completed the AHA-approved BLS course. All participants were enrolled through wide announcements at the hospital and given written informed consent. Additionally, participant data including age, height, BMI, occupation, and work experience were recorded.

### Trial protocol and intervention

As a crossover design, each participant planned to perform three methods (SS = A, KS = B, and KK = C) which random sequences resulting in three periods. Finally, six patterns were generated: ABC, BAC, CBA, CAB, ACB, and BCA.

**Figure 1:**
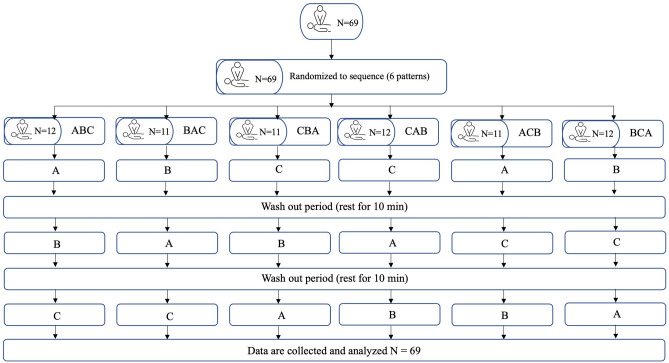
Flowchart of the study. A: stand-on-a-stool (SS), B: stand on stool and one knee kneeling on a stretcher (KS), C: kneeling on the kneeling stool (intervention) (KK).

All participants were randomly assigned to do a different sequence (6 sequences) for three postures (Fig. 1). We considered the risk of a carry-over effect from the previous period. Based on previous study of different resting methods in hand-only CPR, 2-min compression followed by 8-min of 10-s pause after 60-s compression was no significant difference in CPR quality indicators, including chest compression depth, compression with a depth of 5 cm or more, and chest compression rate. Additionally, physiological measures of rescuer fatigue, including heart rate, blood pressure, and pulse pressure, showed no difference^12^. In our study, participants only performed chest compressions for two minutes instead of the ten minutes as in the aforementioned study. This meant that a ten-minute break was sufficient for resting and avoiding any carry-over effects. Thus, we planned that each participant did the chest compressions for two minutes, then took a break of at least ten minutes as a washout period. Furthermore, repeat until all three poses are completed. Quality of chest compression data was obtained by the ALIVE BLS manikin® (Siamtool Engineering Co., Ltd.), capable of measuring compression depth, rate, recoil, and chest compression fraction. Participants answered the post-test questionnaire about preferability, satisfaction and modified Borg’s scale score.

**Figure 2:**
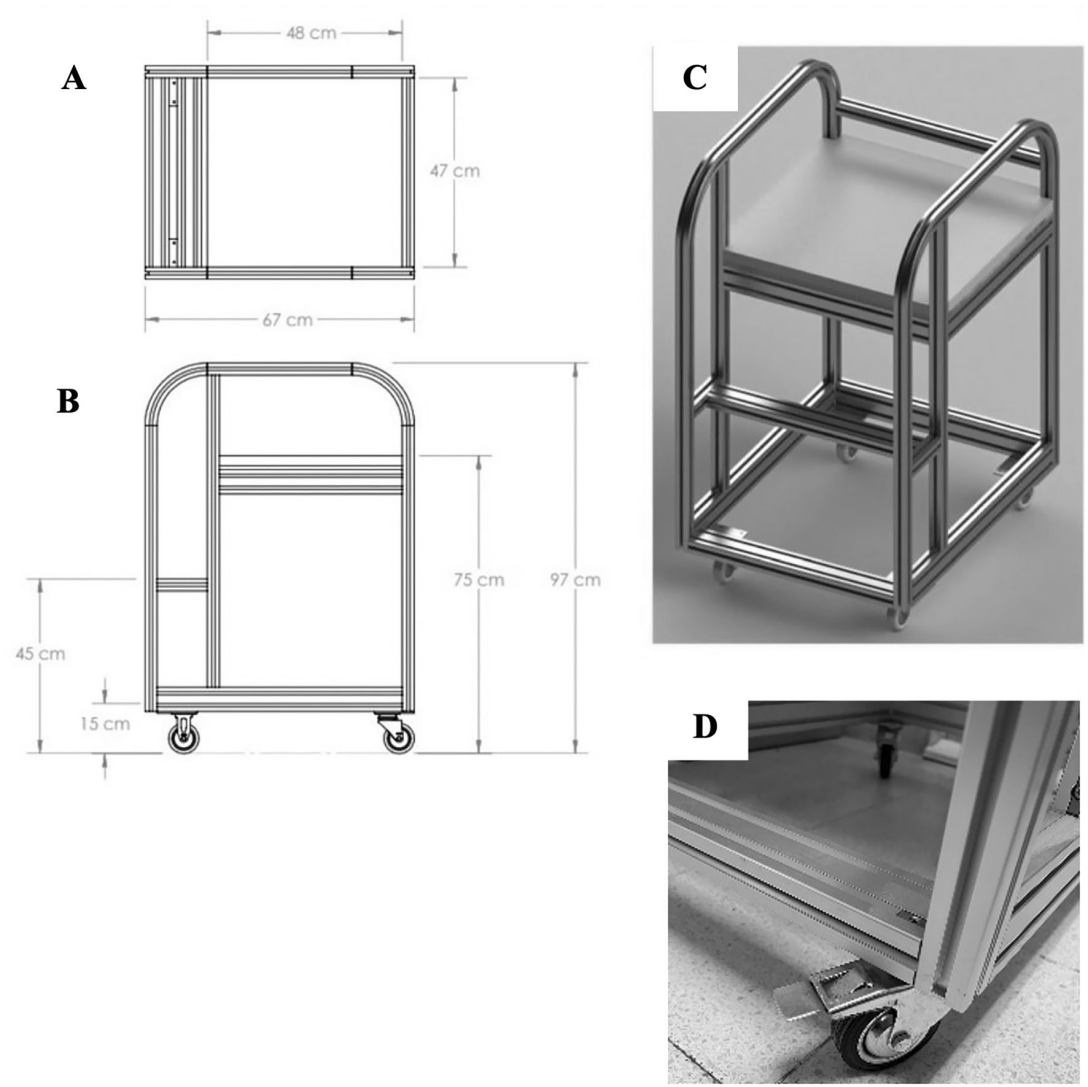
The innovative material of the kneeling stool for the rescuer used to kneel at the same height as the bed. (**A**) Innovation blueprint from above view, (**B**) side view, (**C**) kneeling stool 3D picture, (**D**) wheel lock.

**Figure 3:**
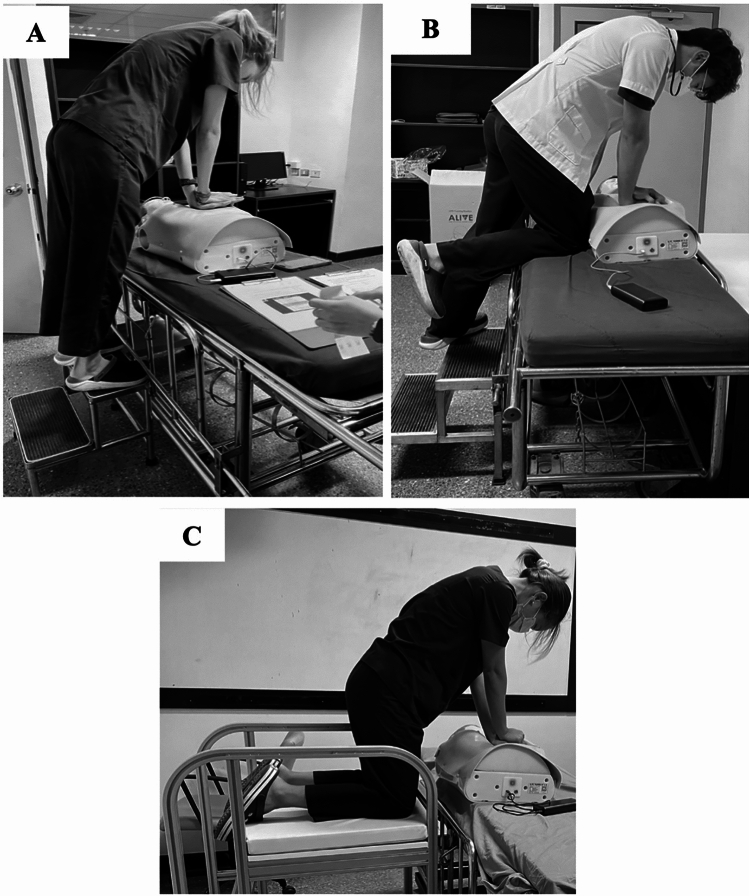
Participants posture. (**A**) Stand-on-a-stool (SS), (**B**) stand on stool and one knee kneeling on a stretcher (KS), (**C**) kneeling on the kneeling stool (intervention) (KK).

For kneeling on a stand at the same height as the patient's bed, our innovation aimed to allow participants to do the chest compressions in a kneeling position beside the patient, which was the kneeling stool. The kneeling stool size was 70 × 80 × 80 cm, with a cushion pad for participant support. The innovative material structure was made of stainless steel (Figs. 2, 3). We limit the height of the bed in the method of SS no more than 20 cm from the knee because a prior study indicated that would reduce the quality of CPR^8^.

### Outcome

High-quality CPR parameters included rate, depth, adequate depth, adequate full chest recoil, and chest compression fraction. So, this study's primary outcome was to compare the effectiveness of chest compression between three methods: rate, depth, adequate depth, and full chest recoil percentage. A secondary outcome was the participant preference, feeling of safety, confidence, and tiredness using the modified Borg's scale score.

### Sample size

The study size estimation was based on the pilot data of 6 BLS-certified rescuers who worked in the ED. All rescuers were randomized in 3 × 3 fashions (three-intervention and three-period), mimicking an actual intended crossover study. Study size estimation via power calculation was done using Monte Carlo simulation of a linear mixed model using parameters estimated from our pilot data dataset. (Supplementary Fig. 1) Three independent components were included in the model: methods (representing the treatment effect), periods (representing the period effect), and the interaction term of methods and periods (representing the sequence effect). We calculated based on adequate chest compression depth parameters in our pilot data that showed method SS, KS, and KK were 80.5 ± 27.1%, 89 ± 23.6%, and 63.8 ± 41%, respectively. Parameters in the linear mixed model for the effect size of 10% were as follows: intercept, method KS’s coefficient, method KK’s coefficient, period 2’s coefficient, period 3’s coefficient, the interaction term of method KS and period 2, the interaction term of method KS and period 3, the interaction term of method KK and period 2, the interaction term of method KK and period 3, random-effect of method parameter, random-effect of period parameter, and observation-level error were 133%, 40%, 69%, − 58%, − 5.5%, 51%, 5%, 70%, − 31%, 51%, 11%, and 38%, respectively. We simulated power for level-2 (number of total patients included) study sizes with an alpha error of 0.05 and set the number of simulations at 1000 replications. Given our assumptions, we estimated that we would achieve more than 90% statistical power to detect the difference in the estimated interaction parameters (the sequence effect) with at least 50 participants. To account for the anticipated 20% of missing data or censored observations, we would include 63 participants.

### Data analysis

Baseline characteristics were summarized as number, percentage, mean, standard deviation, median, and interquartile range (IQR) as appropriate. Data visualization demonstrated the normal distribution of continuous variables, and the categorical variable was determined using Fisher’s exact test. To account for correlated data in a crossover design, we used multilevel models with random effects incorporated with an interaction term of methods and periods to compare the difference of continuous variables among the three methods. If missing data were observed, multiple imputation methods would be used to handle the missing data before proceeding to regression analysis. The Stata version 16 (Stata Corp LLC, College Station, TX, USA) was used to analyze all statistical data. Statistical significance was specified at two-sided p < 0.05.

### Ethics approval

Research Ethics Committee of the Faculty of Medicine, Chiang Mai University approved the research protocol on 6 Sep 2021 (study code: EME-2564-08366).

## Result

A total of 69 participants were eligible for inclusion The mean age of the study participants was 29.5 ± 7.6 years, and there were 51 females (73.9%). The mean weight and height were 60.6 ± 14.8 kg, and 163.6 ± 7 cm, respectively (Table 1). There is no missing data.

**Table 1:** Baseline characteristics.

Characteristics	Overall (n = 69)
Age—years*	29.5 ± 7.6
Female—n (%)	51 (73.9)
Weight—kg*	60.6 ± 14.8
Height—cm*	163.6 ± 7
Body mass index (BMI)—kg/m^2^*	22.6 ± 5
Occupation—n (%)	
Nurse	32 (46.3)
Physician	26 (37.6)
Paramedic	3 (4.3)
Nurse aid	8 (11.5)
Experience in the ED—year^†^	3 (1, 10)

*ED* emergency department, *cm* centimeter, *kg* kilogram.

*Mean ± standard deviation.

^†^Median (interquartile range).

The three methods had no statistically significant differences in adequate chest compression depth (Table 2). In a group of compression depth of less than 5 cm, mean differences (MD) of KK vs SS, KS vs SS, and KK vs KS were − 4.6 cm (95% CI − 17.2 to 7.9 cm, p 0.468), 5.7 cm (− 6.6 to 18 cm, p 0.362), and − 10.4 cm (− 22.9 to 2.2 cm, p 0.105), respectively. In a group of compression depth of 5–6 cm, mean differences of KK vs SS, KS vs SS, and KK vs KS were 5.6 cm (95% CI − 6.7 to 17.8 cm, p 0.372), − 3 cm (− 15.2 to 9.2 cm, p 0.625), and 8.6 cm (− 3.6 to 20.8 cm, p 0.167), respectively. In a group of compression depth of greater than 6 cm, mean differences of KK vs SS, KS vs SS, and KK vs KS were 0.2 cm (95% CI − 11.8 to 12.2 cm, p 0.978), − 4 cm (− 15.9 to 8 cm, p 0.516), and 4.1 cm (− 7.9 to 16.1 cm, p 0.502), respectively. For compression rate, KS vs. SS (MD 2.6 times/min, 95% CI − 2.7 to 7.9 times/min, p 0.332) and KK vs. KS (MD 2.7 times/min, − 2.6 to 8 times/min, p 0.321) were no evidence of difference. KK had a different trend but was not better than SS for chest rate (MD 5.3 times/min, 0 to 10.6 times/min, p 0.05). For adequate full chest recoil percentage, KK was statistically better than SS (MD 22.6%, 7.7 to 37.6%, p 0.003). Also, KS was better than SS (MD 37.2%, 21.8 to 52.6%, p < 0.001). However, there were no statistical differences between the three methods in the modified Borg’s scale score. Based on the satisfying score, the rescuer preferred when performing KK to SS (MD 1.4 points, 0.4 to 2.4 points, p 0.007) or KS (MD 1.4 points, 0.3 to 2.5 points, p 0.012).

**Table 2:** Quality of chest compression in three methods.

	KK	KS	SS	KK vs SS		KS vs SS		KK vs KS	
				Mean difference (95% CI)^†^	p-value*	Mean difference (95% CI)^†^	p-value*	Mean difference (95% CI)^†^	p-value*
Compression rate—times/min									
Mean ± SD	112.4 ± 12.2	112.5 ± 11.8	110.1 ± 13.4	5.3 (0 to 10.6)	0.05	2.6 (− 2.7 to 7.9)	0.332	2.7 (− 2.6 to 8)	0.321
Median (IQR)	111.9 (106.6, 119.2)	112.9 (106.9, 118.4)	111.9 (103.1, 118.2)						
Compression depth—%									
< 5 cm									
Mean ± SD	30.6 ± 39.6	38.2 ± 40.9	29.4 ± 39	− 4.6 (− 17.2 to 7.9)	0.468	5.7 (− 6.6 to 18)	0.362	− 10.4 (− 22.9 to 2.2)	0.105
Median (IQR)	9.7 (0, 70.6)	20.5 (0.8, 91.5)	4.9 (0, 62.4)						
5–6 cm									
Mean ± SD	29.5 ± 27	26 ± 25.3	28.8 ± 29.4	5.6 (− 6.7 to 17.8)	0.372	− 3 (− 15.2 to 9.2)	0.625	8.6 − .6 to 20.8)	0.167
Median (IQR)	25.1 (3.3, 53.3)	19.6 (2.5, 50.7)	16.9 (1.6, 52.3)						
> 6 cm									
Mean ± SD	39.8 ± 38.8	35.9 ± 39	41.8 ± 41.3	0.2 (− 11.8 to 12.2)	0.978	− 4 (− 15.9 to 8)	0.516	4.1 (− 7.9 to 16.1)	0.502
Median (IQR)	30.6 (0, 74.9)	20.5 (0, 78.7)	26.1 (0.5, 92.7)						
Adequate compression depth—%									
Mean ± SD	29.5 ± 27	25.6 ± 25.4	29 ± 29.2	6 (− 6.3 to 18.3)	0.338	− 2.7 (− 15 to 9.6)	0.667	8.7 (− 3.6 to 20.9)	0.165
Median (IQR)	25.1 (3.3, 53.3)	18 (2.2, 50.7)	16.9 (2, 52.3)						
Combine chest compression depth (5–6 cm and > 6 cm)—%									
Mean ± SD	69.4 ± 39.6	61.9 ± 40.9	70.6 ± 39	4.6 (− 7.9 to 17.2)	0.468	− 5.7 (− 18 to 6.6)	0.362	10.4 (− 2.2 to 22.9)	0.105
Median (IQR)	90.3 (29.4,100)	79.5 (8.5, 99.2)	95.1 (37.6,100)						
Adequate fully chest recoil—%									
Mean ± SD	50.1 ± 37.4	58.8 ± 38.5	14.1 ± 25.4	22.6 (7.7 to 37.6)	0.003	37.2 (21.8 to 52.6)	< 0.001	− 14.5 (− 31.8 to − 2.7)	0.099
Median (IQR)	53.2 (13.2, 88.6)	65.9 (18.7, 93.3)	1.6 (0, 14.8)						
Modified Borg’s scale score—score^‡^									
Mean ± SD	2.9 ± 1.6	3.5 ± 1.6	3.1 ± 1.8	− 0.7 (− 1.6 to 0.1)	0.095	− 0.1 (− 1 to 0.8)	0.787	− 0.6 (− 1.5 to 0.3)	0.161
Median (IQR)	3 (2, 4)	3 (3, 4)	3 (2, 4)						
Rescuer’s satisfying score—score									
Mean ± SD	8.2 ± 1.5	6.8 ± 2	7.7 ± 2.1	1.4 (0.4 to 2.4)	0.007	0 (− 1.1 to 1.1)	0.994	1.4 (0.3 to 2.5)	0.012
Median (IQR)	9 (7, 9)	7 (6, 8)	8 (7, 9)						
Rescuer’s preference—n (%)	37 (53.6)	10 (14.5)	22 (31.9)						

*IQR* interquartile range, *SD* standard deviation.

Group KK (group C) = Kneeling on the kneeling stool (Intervention).

Group KS (group B) = Stand on stool and one knee kneeling on stretcher.

Group SS (group A) = Stand-on-a-stool.

*p < 0.05 was statistical significance.

^†^Analyzed with multilevel models with random effects due to crossover design.

^‡^Although the modified Borg’s scale score could be a decimal of 0.5 points, the mean difference might be other decimal points to represent a different trend.

## Discussion

This study demonstrated that a rescuer could perform high-quality CPR using any chest compression method if the rescuer's knee height was equal to the level of the patient's bed. However, the SS method has some limitations in fully recoiling. As in the previous study, it is unclear whether this was due to an incomplete recoil; we suspect it was caused by an unstable position^13,14^. Also, our study found no statistically significant difference in adequate chest compression depth (percentage). Although KK tended to have a slightly higher chest compression rate than SS, compression rates among all methods were still within the recommended range. KK and KS increased the adequate chest recoil percentage, whereas SS produced a surprisingly low rate of adequate chest recoil.

A previous study revealed that the efficiency of chest compression was diminished when the bed was 20 cm higher than the knee of the rescuer^8^. However, this depends on the stepstool and the rescuer's height^15^. Chest compression on a step stool (stand-on-a-stool or SS) demonstrated effective quality in chest compression depth and was not inferior to floor chest compression^7^. Our study discovered that adequate chest compression was achieved when the rescuer's knee was at the same level as the patient's bed; however, the SS approach cannot adjust the level of the rescuer's knee to the bed level, resulting in insufficient chest recoil. Chest recoil is essential to high-quality CPR because it allows more blood to refill the heart to fill between chest compressions adequately. Following OHCA, the ideal Chest compression rate and chest compression dept combination are linked to a positive neurologic outcome^3^.

Our study examined the impact of rescuer position on chest compression quality in a controlled experimental setting. However, real-world CPR scenarios involve additional factors such as compressor changes and interrupted for rhythm analysis, defibrillation, and breathing (in cases without intubation), which contribute to low chest compression fraction or "no flow time" aspects not addressed in our study. While a high ratio in chest compression fraction is crucial for CPR quality, our study did not explore how different CPR positions might affect this aspect. Future research should consider these real-world factors to better understand their impact on CPR quality.

Kneeling CPR is the typical technique provided in BLS courses worldwide while standing CPR is unfamiliar and may initially result in inferior quality. Our research also indicated that standing CPR was associated with poorer quality. In real-world practice, we, therefore, proposed using a kneeling stool. Chest compression on the floor compared to kneeling on the patient’s bed is not different^16–18^. One study suggests that kneeling was the optimal position for CPR since it optimized the time the chest was compressed effectively and reduced back pain^10^. From our post-test questionnaire, most participants preferred the KK because this posture felt stable and familiar, like kneeling on the floor, and they believed that it reduced low back pain compared to the standing posture. If the kneeling stool (KK) was available, most participants preferred to try it. Comparable to the benefit, there may have some disadvantages, including an impediment when changing the rescuer's position, limited space to perform some procedures, and the inability to adjust the height. But our study suggests that three methods may be used to achieve high-quality CPR. Still, we recommend KK and KS because both methods were better at achieving adequate full chest recoil when compared with SS. If CPR is prolonged for more than 2 min, kneeling is better than stand-on-a-stool posture^10^.

## Limitation

This study had some main limitations. First, the primary outcome was chest compression depth while, the manikin could only report rate of adequate depth of compression (less than, greater than or equal to 5–6 cm) which was not the actual depth of the compression. Second, our study utilized manikins which may have affected the rescuer's sensation as it may not have felt the same as when performing chest wall compressions with real people. Furthermore, due to being simulated, the experience gave no stress and tension unlike the real-life situations. Therefore, scenario-based practice and simultaneous data collection of high-quality chest compression may be used in further study.

Third, we estimated the sample size to account for the percentage of adequate chest compression depth. However, there were many CPR quality indicators. Future research may consider calculating the sample size based on various effectiveness endpoints of chest compressions, including considering multiplicity from the perspective of multi-group comparisons. Fourth, we designed a washout period of at least ten minutes between each period to allow participants to take a break before moving on to the next period based on a previous study of 2-min compression followed by an 8-min 10-s pause after 60-s compression that revealed no significant difference in CPR quality indicators. The duration of the washout period varied for each participant but was not recorded. Thus, the duration between periods could be recorded in the future study. In addition, it may be considered if the washout period is lengthened to several hours or days. Lastly, Our study did not address real-world CPR situations involving factors such as changing compressors every 2 min, interrupted compressions for rhythm analysis, defibrillation, providing breaths (in cases without intubation), and during intubation, which may contribute to no-flow time.

## Conclusion

The three methods had no statistically significant differences in adequate chest compression depth (percentage). Still, both kneeling on the kneeling stool (KK) and one knee kneeling on a stretcher (KS) achieved better adequate chest recoil, so we recommend using these two methods for high-quality CPR in the ED.

### Supplementary Information

Supplementary Figure 1.

## Data Availability

The datasets are available from the corresponding author upon reasonable request.
