# Supplementary figures and images for: Effectiveness of innovative chest compression on the emergency department stretcher by an alternative method: a randomized controlled crossover trial
Source: Sci Rep. 2024 May 29;14:12284. doi: 10.1038/s41598-024-62845-y (PMC11137072; doi:10.1038/s41598-024-62845-y)

**Supplementary data**


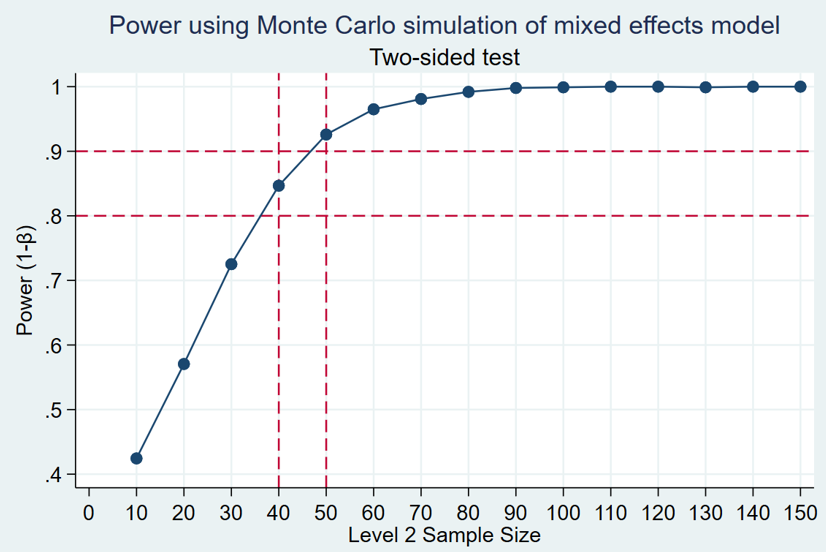


**Supplementary figure 1 .** Monte Carlo simulation for study size estimation

Supplement: Supplementary file 1 — Supplementary Figure 1. [file 41598_2024_62845_MOESM1_ESM.docx]
